# Cost-effectiveness of preemptive skin treatment to prevent skin-toxicity caused by panitumumab in third-line therapy for KRAS wild type metastatic colorectal cancer in Japan

**DOI:** 10.1186/s40780-021-00218-7

**Published:** 2021-10-01

**Authors:** Munenobu Kashiwa, Ryo Matsushita

**Affiliations:** Faculty of Pharmacy, Institute of Medical, Pharmaceutical and Health Sciences, Kanazawa University, Kakuma-machi, Kanazawa, 920-1192 Japan

**Keywords:** Panitumumab, Skin-toxicity, Cost-effectiveness, Colorectal cancer

## Abstract

**Background:**

Clinical management of skin-toxicity associated with the use of anti-Epidermal Growth Factor Receptor (EGFR) antibodies to treat colorectal cancer maintains quality of life of patients with colorectal cancer. Results of clinical trials have recommended the efficacy of prophylactic treatment, but the cost-effectiveness is unclear. This study examined the cost-effectiveness of preventive skin care for skin-toxicity caused by panitumumab in third-line therapy for KRAS wild type metastatic colorectal cancer from the perspective of the Japanese healthcare payer.

**Methods:**

The data source was J-STEPP trial, which compared preemptive skin treatment with reactive treatment in third-line panitumumab therapy for KRAS wild type metastatic colorectal cancer in Japan. The costs and effectiveness of preemptive treatment was compared with reactive treatment in a 3-year time horizon using a 4-state partitioned survival analysis. The health outcome was quality-adjusted life-years (QALYs). The costs were 2020 revisions to the drug prices. The robustness of the model was verified by one-way sensitivity analysis and a probabilistic sensitivity analysis (PSA). A 2% annual discount was applied to the expenses and QALYs. Willingness-to-pay (WTP) threshold of 5 million JPY was used.

**Results:**

Preemptive treatment had incremental effects of 0.0029 QALYs, incremental costs of 5300 JPY (48.6 USD), and incremental cost-effectiveness ratios (ICER) of 1,843,395 JPY (16,912 USD) per QALY. The variability of preemptive and reactive treatment costs for skin-toxicity and the disutility of skin-toxicity had a large impact on ICER. From PSA, the cost-effectiveness rate of preemptive treatment was 75.0%.

**Conclusions:**

The cost to effectiveness of preemptive treatment to prevent skin-toxicity caused by panitumumab in third-line therapy for KRAS wild type mCRC is not high.

## Background

Colorectal cancer is the fourth most common malignancy in the world and the third leading cause of cancer-related deaths [1]. In Japan, colorectal cancer is the second leading cause of death after lung cancer, and more than 50,000 people died of it in 2017 [2]. The chemotherapy for unresectable colorectal cancer has made remarkable progress in the last decade. Those with a median overall survival (OS) of 11 to 12 months in the era of 5-FU single-agent or 5-FU/LV combined administration have recently survived around 30 months [3]. It has become possible to think that the extension of this survival period is mainly due to the fact that the treatment can be continued, not only the primary treatment, but also the second-line and later treatments due to the emergence of new drugs and molecularly targeted drugs. Anti-Epidermal Growth Factor Receptor (EGFR) antibodies are widely used in patients for RAS wild type metastatic colorectal cancer (mCRC) from first-line therapy to salvage lines [4, 5]. This drug is a major concern for clinicians because of the skin-toxicity that occurs in almost all patients [6, 7]. Skin-toxicity caused by anti-EGFR antibodies can lead to acne-like dermatitis, paronychia, and dry skin, with deleterious effects on the patient’s quality of life. Treatment that focuses primarily on preventing serious symptoms of skin-toxicity is important [8, 9]. Preemptive skin treatment is an effective strategy for preventing skin-toxicity with anti-EGFR antibodies. Skin-toxicity evaluation protocol with panitumumab (STEPP) study showed that preemptive skin treatments such as sunscreens, skin moisturizers, topical steroids, and doxycycline are effective in preventing the development of grade 2 or higher skin-toxicity in the United States [10]. In another similar study, Japanese Skin-toxicity Evaluation Protocol with Panitumumab (J-STEPP), involving only Japanese patients, preemptive skin treatments such as sunscreen, skin moisturizers, topical steroids, and minocycline was effective in preventing the development of skin-toxicity in third-line Panitumumab (Pmab) therapy for KRAS wild type mCRC [11]. Based on these results, preemptive skin treatment is recommended over reactive skin treatment [12, 13]. It has been reported that preemptive skin treatment is widely used in real world clinical settings [14]. However, the cost-effectiveness of preventive treatment is unclear. Since health economics is now an important issue to focus on, it is important to consider not only whether statistical differences are clinically significant, but also whether they are clinically significant in terms of health economics. The purpose of this study was to estimate the cost-effectiveness of a preemptive skin treatment for skin toxicity in third-line Pmab therapy for KRAS wild type mCRC compared to a reactive skin treatment from the perspective of the Japanese health insurance system payer.

## Methods

### Treatment strategies

The cost-effectiveness of 2 skin treatment strategies from the perspective of the Japanese healthcare payer was compared.

Strategy 1: Preemptive treatment consisted of skin moisturizer, sunscreen, topical steroid and minocycline 100 mg per day.

Strategy 2: Reactive treatment consisted of only skin moisturizer (and sunscreen if patients requested).

Skin moisturizers and topical steroids were applied to the face, hands, feet, neck, back and chest in the morning and evening (bedtime). The application of moisturizers and topical steroids twice a day was chosen to avoid potential patient confusion due to the application of each once a day, and because of the evening bathing habits of Japanese patients. Sunscreen was applied to the exposed area before going outside. Minocycline 100 mg/day was administered.

### Clinical Data

The data source was the result of J-STEPP trial in Japan [11]. In this trial, patients had KRAS wild type mCRC with evaluable disease, and two prior chemotherapy regimens for mCRC (adjuvant chemotherapy with fluoropyrimidine alone was not included in the count of primary treatment if disease recurrence occurred during or within 6 months from completion). Other key eligibility criteria were age ≥ 20 years, Eastern Cooperative Oncology Group performance status score ≤ 2, and adequate organ function. Patients were excluded if they had received prior anti-EGFR therapy.

Pmab was administered at 6.0 mg/kg every 2 weeks. If ≥grade 3 skin-toxicity occurred, administration of Pmab was suspended, and Pmab dose reduction was performed after recovery to ≤grade 2. The chemotherapy regimen was chosen by each investigator. There were 48 cases in the reactive treatment arm and 47 cases in the preemptive treatment arm. At 6 weeks after the start of the study, the incidence of grade 2 or higher skin-toxicity, as determined by the Central blinded review of dermatologist, was 50.0% in the reactive arm and 18.6% in the preemptive arm (Risk Ratio = 0.37, 95% Confidence Interval (CI): 0.19–0.74, *p* = 0.002). Median OS were 8.2 months (95% CI: 5.8–13.1) in the preemptive arm and 12.1 months (95% CI: 6.7–21.7) in the reactive arm (Hazard Ratio (HR): 1.19, 95% CI: 0.75–1.90, log-rank: *p* = 0.469), and median progression-free survival (PFS) were 3.6 months (95% CI: 2.4–4.9) and 4.0 months (95% CI: 2.8–4.5), respectively (Hazard Ratio: 1.20, 95% CI: 0.78–1.84, log-rank: *p* = 0.413), with no significant difference between the 2 arms. For this analysis, Grade 3 or higher skin-toxicity was defined as severe. The results of time to skin-toxicity, PFS, OS, and rates of severe skin-toxicity from the J-STEPP trial were used for this analysis. Time to skin-toxicity was used from the results of each group, while PFS and OS were used from the combined results of both groups. The incidence of skin toxicity in Japanese clinical trial was 98% in almost all patients [6]. The Dermatology Life Quality Index showed that skin toxicity was distressing to patients. The skin toxicity was reversible but persisted for a long time, with a median duration of 4.97 months. Therefore, a partitioned survival model to its skin toxicity was applied. In this analysis, parametric models of the skin toxicity occurrence curves were extrapolated. It was assumed that skin toxicity appeared along the models, that there was quality of life decrement due to the skin toxicity, and that skin treatment was continued until PFS after the appearance of skin toxicity. It was defined that there was no expression of the side effect by the skin treatment and that there was no effect on utility value and cost, since the trial had no adverse events associated with the assigned skin treatment regimen and no treatment-related deaths.

### Disease modeling

**Fig. 1:**
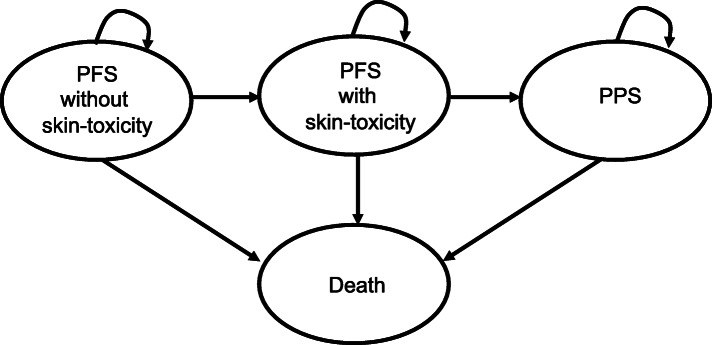
Partitioned survival model. PFS: Progression-free survival, PPS: Post-progression survival

**Fig. 2:**
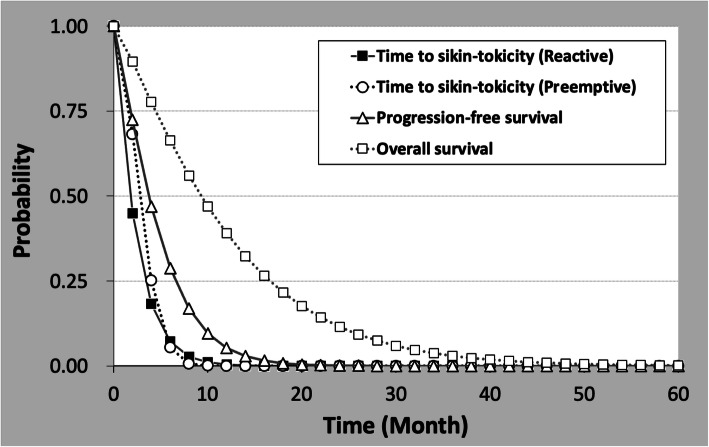
Model curves

For this analysis, a partitioned survival model was constructed with 4-states. A partitioned survival model is commonly used in late-stage oncology modeling [15]. In our approach, we modeled the prognosis of cancer patients with four states: progression-free survival (without skin toxicity), progression-free survival (with skin toxicity), post-progression survival, and death, and estimated the health care cost, years of survival, and QALY of the patients. For each state, QALY and cost per cycle are established. (Fig. 1) The inward arrow indicates that it stays in the same state per unit period. Parametric functions were fitted to data on time to skin toxicity, progression-free survival, and overall survival from clinical trials. The fitted parametric function was then used to calculate the change in the proportion of patients in each condition over time. Expected values of cost and QALYs were calculated by combining the calculated percentage of patients with cost and QOL values for each state. Costs associated with adverse events and changes in QALY are incorporated into calculations as expected value. The Kaplan-Meier curves were digitized from the literature and the individual patient data were reconstructed and curve-fitted. The curve-fitting functions were determined according to the Akaike information criterion and visual plausibility. Exponential, log-logistic, log-normal, Weibull, and gamma distributions were considered as model curves. Weibull curves were extrapolated to fit to Kaplan-Meier survival curves. (Fig. 2) The scale (λ) and shape (γ) parameters were determined using the method for estimating the underlying survival distribution from Kaplan-Meier curves. OS is a clinical parameter that includes death unrelated to colorectal cancer. Therefore, background mortality was not included in the simulation. A 5-year time horizon was used in the model (ie, costs and outcomes for patients were considered up to 5 years after the initiation of treatment). This time horizon was assumed to reflect patient lifetime, since the model predicted that, by this time, over 99% of patients had died in each treatment arm, and further extrapolation of model results was deemed unnecessary for decision-making. In this model, treatment for skin-toxicity was defined as continued from the PFS with skin-toxicity phase to the period of skin-toxicity improvement in PPS. The proportion of serious skin toxicity was used to fit the cost and utility values of serious skin toxicity.

### Costs

Costs were estimated from the perspective of the healthcare payer, therefore, only direct medical costs were included. There was not enough information from the clinical trial on additional cost such as medical care of dermatologist in the skin-toxicity onset. In this model, preemptive skin treatment was defined as outpatient management by a chemotherapist and skin treatment after the onset of skin-toxicity was managed by a dermatologist. The medical costs considered in this model were drug costs and medical fees for skin treatment. (Table 1) The costs related to outpatient chemotherapy, pharmacy costs, adverse events other than skin treatment were not included in this analysis because they would be similar in both groups. In this model, skin-toxicity treatment was defined as continuing until PFS, and post-PFS skin treatment costs were assumed to be similar for both groups and were not included in the calculations. The severe skin-toxicity rate of both groups was extracted, and the skin treatment prescription was separately set.

**Table 1:** Variables

Variables	Base case	Range	Distibution	Reference
Preemptive skin treatment cost	5324 JPY (48.8 USD)	50%	Gammma	11, 14, 15, 16, 17, 18
Treatment cost for skin-toxicity	16,460 JPY (151.0 USD)	50%	Gammma	11, 14, 15, 16, 17, 18
Treatment cost for severe skin-toxicity	20,520 JPY (188.3 USD)	50%	Gammma	11, 14, 15, 16, 17, 18
Rate of severe skin-toxicity (Preemptive)	0.023	50%	Beta	11
Rate of severe skin-toxicity (reactive)	0.114	50%	Beta	11
Duration of skin treatment in post-progression state	41 days	95%CI	Normal	MHLW application for approval
Utility for Progression-free survival state	0.680	30%	Beta	20
Utility for Post-progression survival state	0.730	30%	Beta	20
Disutility for skin-toxicity	0.033	50%	Beta	21, 22, 23, 24
Disutility for severe skin-toxicity	0.100	50%	Beta	21, 22, 23, 24
Discount rate	0.02	0–0.04		27

1 USD = 109.0 JPY (2019 annual exchange rate)

The cost of preemptive treatment was calculated from the drug prices of medium-rank hydrocortisone cream, moisturizing ointment, and minocycline tablets. Reactive treatment did not include skin treatments. Very strong betamethasone propionate, moisturizing ointment, and minocycline tablets were used for the onset of skin-toxicity. Strongest dexamethasone clobetasol propionate was used for the severe skin-toxicity.

It was assumed that branded medicines were given to patients. The skin treatment prescription content was set based on previous reports [14, 16]. Costs were calculated according to 2020 medical fees and drug tariff in Japan [17, 18]. The costs calculated in Japanese yen (JPY) were converted to US dollars (USD) using the yearly exchange rate of 2019 announced by the OECD (1 USD = 109.0 JPY) [19].

### Health-related utility

The primary measures of effectiveness in this analysis was quality adjusted life-years (QALYs) gained. To estimate the total QALYs in the model, survival time was adjusted by QALYs. The utility values used in the base case analysis were taken from previously published literature, because the utility value in Japanese K-RAS wid type mCRC patients in third-line Pmab therapy has not been reported. EuroQol 5 Dimension utilities were reported as 0.73 (Standard Deviation (SD): 0.24) and 0.68 (SD: 0.23) for K-RAS wild type patients who received Pmab plus Best supportive care (BSC) and BSC alone, respectively [20]. Utility values of 0.73 and 0.68 were used in patients with PFS without skin-toxicity and PPS, respectively. In this analysis, the differences in quality of life decrement due to skin-toxicity in QALYs were compared. The disutility was applied to the decrement of quality of life. The disutility values used in the base case analysis were taken from previously published literature. The disutility of skin-toxicity and severe skin-toxicity in the base case were extracted as − 0.033 and − 0.10 from the published literature, respectively [21–24]. Skin-toxicity occured to varying degrees in almost all patients treated with Pmab and is a troubling side effect [6]. Though the skin treatment is actually carried out because it is expected to prevent the decrement of the quality of life, the quantitative evaluation which shows the decrement of the quality of life by the skin-toxicity is not consistent [9, 25]. Therefore, in this analysis, the effect by the fluctuation of the disutility was examined widely in sensitivity analysis. The period until the skin-toxicity improved after the end of Pmab therapy was defined as a period in which the disutility occurred and the treatment cost was necessary. In the base case, the period until improvement of skin-toxicity was set at 41 days from the application data for approval review of Pmab.

### Cost-effectiveness analysis

Cost-effectiveness was evaluated using the incremental cost-effectiveness ratio (ICER), the ratio between cost increments and QALY increments. In this analysis, the willingness-to-pay (WTP) threshold was set at 5 million JPY/QALY based on a commonly used threshold [26, 27]. A base case analysis that incorporated the baseline parameters was performed. The costs and QALYs were discounted at a rate of 2% per annum in the base case analysis, based on the Guideline for the Economic Evaluation of Healthcare Technologies in Japan [27].

### Sensitivity analysis

Several sensitivity analyses were performed to evaluate the uncertainty and robustness of the model. For these sensitivity analyses, the parameters were selected to cover all potential areas of uncertainty, such as the survival curves for Time-to-skin-toxicity and PFS, drug costs, and disutility values. One-way sensitivity analysis assessed the impacts of varying model parameters on the ICER. The costs and disutilities fluctuated within ±50%. The utilities for PFS and PPS were varied 30%. The period of skin-toxicity in PPS was varied within a range of the 95% confidence interval. The discount rate was varied from 0 to 4%. A probabilistic sensitivity analysis was also performed to assess the impact of sensitivity on the model parameters using Monte Carlo simulation with 10,000 samples. By plotting the large amount of data obtained from the simulation on an incremental cost-effectiveness plane, we can show the probability of acceptance of the intervention for WTP. The standard normal distribution was used for the Weibull parameters, the gamma distribution was used for the cost parameters, and the beta distribution was used for the disutility parameters. For each run of the simulation, input values for the parameters were drawn at random from appropriate distributions. The Weibull parameters of the curves for Time to skin-toxicity and PFS were generated using the variance-covariance matix [28]. (Table 2) All of the analyses were performed using TreeAge Pro software version 2020 (TreeAge, Williamstown, MA).

**Table 2:** Model parameters

Parameters	Base case	Standard error	95% Confidence interval	
Time to skin-toxicity (Preemptive)				
Shape	1.851	0.216	1.427	2.275
Scale	0.106	0.037	0.034	0.178
Time to skin-toxicity (Reactive)				
Shape	1.082	0.120	0.847	1.318
Scale	0.379	0.084	0.215	0.543
Progression-free survival				
Shape	1.233	0.092	1.054	1.413
Scale	0.137	0.029	0.081	0.194
Overall survival				
Shape	1.198	0.967	1.009	1.388
Scale	0.479	0.143	0.020	0.076

## Results

### Base case results

The base case model results are presented in Table 3. Compared with reactive treatment, preemptive treatment was associated with a gain of 0.0029 QALYs. The incremental cost was 5300 JPY (48.6 USD) in 5 years. Therefore, the ICER per QALY was 1,843,396 JPY (16,912 USD).

**Table 3:** Base case result

	Total Cost JPY (USD)	Incremental Cost JPY (USD)	QALYs	Incremental QALYs	ICER JPY/QALYs (USD/QALYs)
Reactive	39,809		0.6726		
	(365.2)				
Preemptive	45,109	5300	0.6755	0.0029	1,843,395
	(413.8)	(48.6)			(16,912)

JPY: Japanease yen, USD: US dollars, QALYs: Qality adjusted life years

1 USD = 109.0 JPY (2019 annual exchange rate)

### Sensitivity analysis

**Fig. 3:**
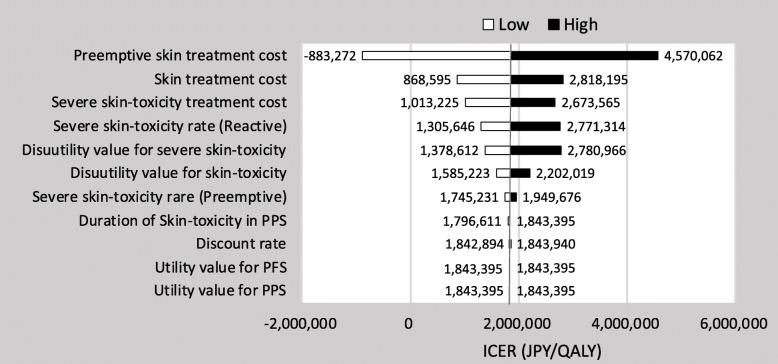
One-way sensitivity analysis. PPS: Post-progression survival, PFS: Progression-free survival, ICER: Incremental cost-effectiveness ratio, JPY: Japanease yen, QALY: Quality adjusted life years

**Fig. 4:**
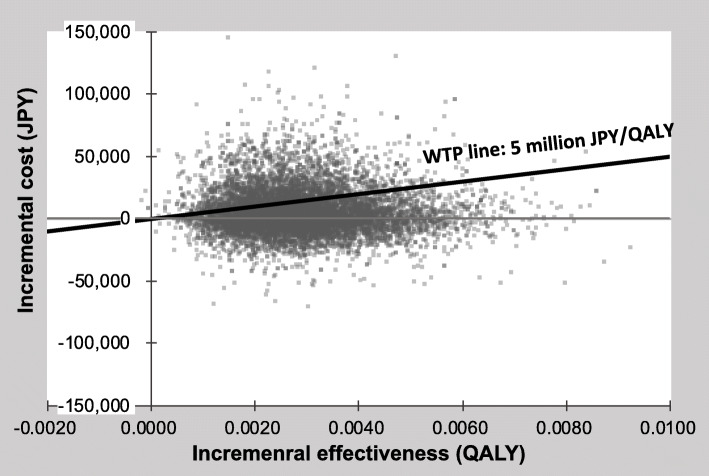
Cost-effectiveness plane. JPY: Japanease yen, QALY: Quality adjusted life years, WTP: Willingness-to-pay

The result of the one-way sensitivity analysis was presented in tornado diagrams (Fig. 3). The parameters with the greatest impact on the ICER was the preemptive skin treatment cost. Fluctuations in the cost of preemptive skin treatment was affected, but not significantly. The variation of disutility for severe skin-toxicity also had significant impact. Across defined variations in the values of each parameter, the ICER remained below 5 million JPY/QALY. The result of the probabilistic sensitivity analysis was shown in the cost-effectiveness plane. (Fig. 4) The cost-effectiveness plane plotted the incremental effectiveness of a treatment strategy (relative to a comparator) against the incremental cost of the treatment strategy. Each point on the plot was from a particular random draw from the PSA. The WTP-line in the plot was the threshold line, with slope equal to 5 million JPY/QALY. For the WTP-line, points below the line were cost-effective while those above it were not. The probability that preemptive treatment would be cost-effective at WTP values of 5 million JPY/QALY was 75.0%.

## Discussion

This present study is the first to demonstrate the cost-utility analysis of preemptive skin treatment compared reactive skin treatment for skin-toxicity caused by Pmab therapy in KRAS wild type mCRC in Japan. The ICER remained below 5 million JPY/QALY even if the conditions were changed. The PSA showed that the probability of preemptive skin treatment being cost-effective was 75.0% at the WTP threshold of 5 million JPY/QALY. Findings from these uncertainty analyses suggest that preemptive skin treatment is cost-effective at commonly accepted thresholds compared with reactive skin treatment.

In the one-way sensitivity analysis, the impacts of the values were examined. In particular, diutility of skin-toxicity has not been found consistently in previous reports [25], and its variation has been analyzed widely. The disutility value used in this study was based on the published previous literature, because no disutility value data in Japanese patients with the same conditions was reported in this analysis. It should be considered that the quality of life decrement actually occurs, though it is a little, because that the skin-toxicity does not affect the patient quality of life becomes that there is almost no merit in the preventive treatment. The result of PSA showed the robustness of the results including such uncertainty, and the recommendation of the preemptive skin treatment was suggested from the medical economic efficiency in addition to the clinical effect.

Pmab therapy is recommended widely from first-line to salvage line treatment in wild type RAS mCRC. Since severe skin-toxicity is one of the criteria for discontinuation and also affects the rate of continued treatment, control of skin-toxicity is important for patient prognosis. Because the J-STEPP trial targeted mCRC patients treated with third-line Pmab therapy, this analysis is only a cost-effective result within the scope. Therefore, it is necessary to verify the effectiveness of preemptive skin therapy for the prevention of skin-toxicity by EGFR inhibitors in the primary and secondary lines and in combination with other drugs. However, if preemptive skin treatment is used in a similar price range, it is unlikely that the ICER will exceed the threshold of 5 million JPY and is expected to be cost-effective.

Yamazaki et al. extracted large-scale clinical data and reported that preemptive skin treatment is widely available when EGFR inhibitors are used in Japan [14]. As a result of preventing severe skin-toxicity, dermatological visits decreased. In this analysis, we assumed that preemptive skin treatment strategy included the same additional cost of dermatologist visits after the onset of skin-toxicity as reactive skin treatment strategy. The cost-effectiveness of preemptive skin treatment could improve further when the cost of a dermatologist for skin-toxicity is decreased.

Masago et al. reported that the Rash management by the team medical treatment for the EGFR inhibitor use case was effective for the prevention of the severe skin-toxicity [29]. Actual cost-effectiveness cannot be achieved without not only prescribing the necessary medications, but also ensuring that the patient continues with appropriate skin care over time. The consistent guidance to the patient is necessary for that. A collaborative system among pharmacists, nurses, and physicians is important for patients to understand the value of preventive skin care, maintain compliance with preventive skin care, and improve treatment outcomes in clinical practice. The collaboration may be key to ensuring that chemotherapists can adequately treat skin conditions and reduce visits to dermatologists.

There are several limitations to our cost-effectiveness analysis. First, the J-STEPP trial used as a data source did not have a large sample size. Actual clinical outcome may not be sufficiently reflected. Accounting for this, sensitivity analyses were widely performed. Although the results of PSA for assessing such uncertainty showed favorable cost-effectiveness for preemptive skin treatment, further approaches to these uncertainties require estimates based on real clinical data. Second, there is uncertainty from model analysis. In this analysis, parametric survival modelling allowed extrapolating survival curves beyond the observed data period. The advantage of this model is that it enables analysis over a long period of time. In the base case, the most appropriate distribution was selected using the processes of assessing the visual relevance of the observed Kaplan-Meier, statistical relevance (measurement by AIC), and the adequacy of long-term extrapolation. There are limitations of this study about uncertainties regarding the statistical methods used to extrapolate data. Third, since there are no health scale data on skin toxicity measured in Japan, we referred to previous studies in foreign countries to set utility values. Although the utility in lung cancer patients cannot be completely determined as skin toxicity due to side effects, this is the only report to measure health scale data for skin disorders. Since this utility value has been used in previous studies and NICE official analyses as an effect due to skin toxicity, the same value was used in this study. Since the utility was supposed to be different by the severity of the skin toxicity, the fluctuation was widely examined by the sensitivity analysis, and there was no dominant effect. With the progress of QOL research in Japanese patients, this analysis can be updated. Forth, in actual clinical practice, patients may not experience skin toxicity while using panitumumab. However, since skin-toxicity was observed in almost all cases in clinical trials, it was applied to the model based on that. The model was set to transition to the next state when the model curves were crossed, thus reproducing no skin toxicity. Since it was very small, it seemed to have little effect on the results.

The present cost-effectiveness analysis showed that the preemptive skin treatment for skin-toxicity caused by third-line Pmab therapy was cost-effective for KRAS wild type mCRC. Further validation of skin treatment is needed based on QOL surveys on skin-toxicity, the efficacy of preemptive skin treatment in primary and secondary Pmab therapy, and the use of other EGFR inhibitors.

## Conclusions

It was shown that the skin preemptive treatment for the Pmab therapy was recommended based on not only from the clinical effect but also cost-effectiveness.

## Data Availability

The datasets used and/or analysed during the current study are available from the corresponding author on reasonable request.

## Abbreviations

CI: Confidence interval
EGFR: Epidermal growth factor receptor
HR: Hazard Ratio
ICER: Incremental cost-effectiveness ratio
J-STEPP: Japanese skin-toxicity evaluation protocol with panitumumab
JPY: Japanese yen
mCRC: Metastatic colorectal cancer
OS: Overall survival
PFS: Progression-free survival
Pmab: Panitumumab
PPS: Post-progression survival
PSA: Probabilistic sensitivity analysis
QALYs: Quality adjusted life-years
STEPP: Skin-toxicity evaluation protocol with panitumumab
USD: US dollars
WTP: Willingness-to-pay
